# Hyporheic Reaction Potential: A Framework for Predicting Reach Scale Solute Fate and Transport

**DOI:** 10.1021/acs.estlett.4c00035

**Published:** 2024-05-06

**Authors:** Kenneth Swift Bird, Alexis Navarre-Sitchler, Kamini Singha

**Affiliations:** † Hydrologic Science and Engineering Program, Colorado School of Mines, Golden, Colorado 80401, United States; ‡ Geology and Geological Engineering Department, Colorado School of Mines, Golden, Colorado 80401, United States

**Keywords:** Surface water-groundwater exchange, redox geochemistry, hyporheic zone, iron fate and transport

## Abstract

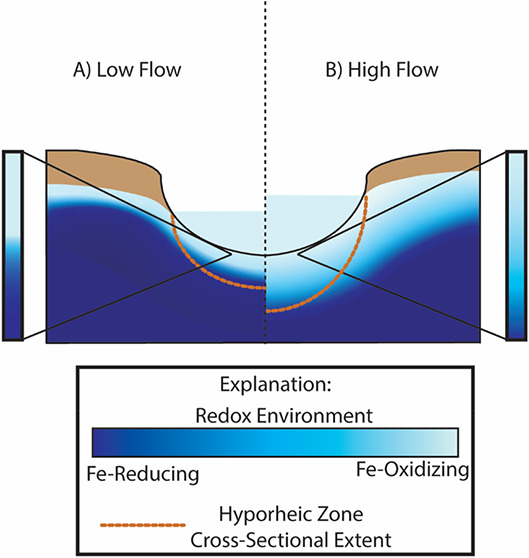

We develop a new framework, hyporheic reaction potential (HRP), to predict the influence of oxidation-reduction reactions on metal fate and transport in streams using data from tracer studies and geochemical sampling. HRP, with energy flux units [KJ m^–2^ s^–1^], is a metric calculated from both the physical and chemical properties of the hyporheic zone. We apply the HRP framework for iron reactions, using existing geochemical and geophysical data from two metal-impacted alpine streams at high and low flow. In these two systems, HRP delineates contrasting controls on iron fate and transport with biogeochemical controls in Mineral Creek and physical controls in Cement Creek. In both systems, HRP scales with discharge and hyporheic-zone extent as flows change seasonally, which demonstrates the ability of HRP to capture physical aspects of chemical reactions in the hyporheic zone. This paper provides a foundation on which HRP can be expanded to other solutes where chemical gradients in the hyporheic zone control reaction networks, making it broadly applicable to redox cycling in stream systems. This framework is useful in quantifying the role of the hyporheic zone in sourcing and storing metal(loid)s under varying hydrologic conditions with implications for water quality, mine remediation, and regional watershed management.

## Introduction

Acid-mine drainage (AMD) is a ubiquitous problem globally, generated from the oxidation of sulfide minerals, which produces low pH, high-metal(loid) concentration waters that affect drinking water quality, aquatic toxicity to plants and animals, negative health outcomes, and bridge corrosion.^1^ Despite the $2.9 billion spent by the U.S. Government alone to mitigate effects of AMD on recreational and municipal waters,^2^ AMD-impacted waterways and watersheds persist, in part because mine-remediation efforts often focus on capture and treatment of point sources flowing from mines, while diffuse sources and oxidation pathways continue to source AMD at the watershed scale.^3,4^ Additionally, groundwater-flow pathways, geochemical composition, reaction rates, and residence times are temporally variable and shift seasonally, which alters groundwater inputs and metal(loid) loading into streams^5−9^ making any remediation strategy difficult to employ over both time and space. Hydrologic events, such as snowmelt runoff and storms, affect the hyporheic zone, the mixing zone at the surface water-groundwater (SW-GW) interface. Hyporheic-zone area defines where biogeochemical work can happen and is controlled by changes in surface water or groundwater elevation.^10−12^

Changes in hydrologic conditions can potentially switch the hyporheic zone from a solute sink to a source^13−15^ as hyporheic zone areas and residence times, redox conditions, and geochemical conditions change,^10,14,16^ affecting carbon, nitrogen, organic compound, and metal cycling^17−20^ at the stream reach and catchment scale. During high-discharge episodic events, like spring snowmelt or intense rains, water infiltration increases dissolved-oxygen (DO) concentrations in the hyporheic zone, the hyporheic zone expands (assuming no bedform mobility), and snowmelt-water pulses may cause mobilization of streambed colloidal metal precipitates.^21,22^ These events temporarily convert the hyporheic zone from a solute sink to a solute source and flush trace metals from the hyporheic zone to the stream in gaining sections of stream.^14,23^ This flushing refreshes the sorption capacity of mineral surfaces to act again as a solute sink once the hyporheic zone contracts as the stream returns to stable base-flow conditions and metal concentrations increase. The conditions that trigger a change in hydrologic and geochemical processes—whether short-term or long-term—and result in the hyporheic zone being a source or sink for metal(loid)s cannot easily be predicted. As climate changes, two important aspects of the hydrologic system are predicted to change: (1) snowpack density and snowmelt timing are expected to become more variable^24^ and (2) metal(loid) concentrations in groundwater are expected to increase as weathering fronts advance deeper in the subsurface due to water table declines.^23,25^ Taken together, these two changes could limit the processing capacity of the hyporheic zone by clogging of hyporheic sediment pores due to mineral precipitation^26^ and prolonged low-flow conditions that limit colloidal transport in the hyporheic zone.^27^ Consequently, quantifying the role of the hyporheic zone in sourcing and storing metal(loid)s under varying hydrologic conditions and the associated impacts remains a key and understudied problem for understanding water quality, mine remediation, and regional watershed management.

Here, we develop a data-driven, process-based framework to quantify event-based fate and transport for redox-sensitive solutes in the hyporheic zone based on a new concept, the hyporheic reaction potential (HRP), by measuring and modeling hyporheic zone characteristics and solute concentrations in groundwater and surface water. This paper presents an example of how HRP can quantify solute fate and transport in the hyporheic zone for iron(Fe)-rich systems and outlines how HRP can be defined for any redox-sensitive reaction.

## Methods and Materials

HRP integrates chemical favorability, SW-GW mixing, and the hyporheic zone area to define overall HRP in the hyporheic zone. We have investigated three common Fe-oxidation pathways that exist in AMD-impacted waters: (1) oxidation of dissolved Fe^2+^ to Fe^3+^ by oxygen, (2) formation of metastable Fe-oxyhydroxy-sulfate schwertmannite that is prevalent in AMD systems, and (3) formation of ferrihydrite, a more stable Fe oxide. Schwertmannite is commonly the first Fe mineral to precipitate in acidic, high-sulfate AMD systems. It has poor crystallinity and over time converts to more stable Fe-(oxy)hydroxides such as ferrihydrite, goethite, and jarosite. We write the oxidized or mineral form on the left and the reduced or soluble form on the right so that HRP is positive when oxidation or precipitation is favored and negative when reduction or dissolution is favored.
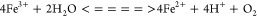
1
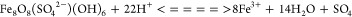
2
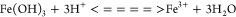
3

**Figure 1:**
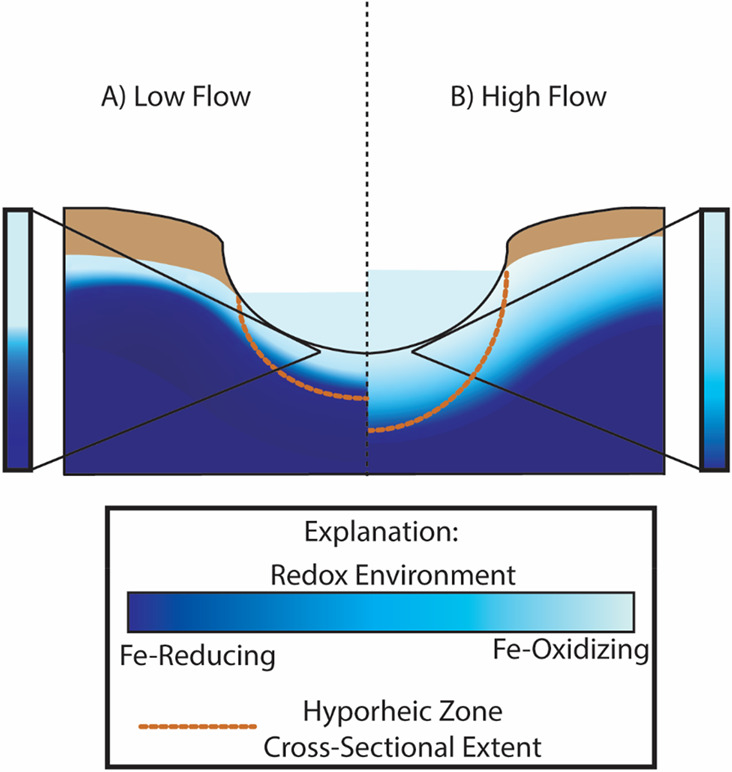
Schematic cross section of the hyporheic zone under (A) low-flow conditions where the area of the hyporheic zone is small and a strong DO gradient persists under groundwater-dominated conditions in the stream, creating a low mixing efficiency, and (B) high-flow conditions where the area of the hyporheic zone expands as stream discharge increases and infiltration of surface water causes a small DO gradient that fosters elevated mixing efficiency.

We define mixing efficiency in the hyporheic zone (ε) [mol m^–4^ s^–1^] as a function of both chemical gradients and physical parameters. For redox-sensitive reactions, DO gradients are expected to control reaction networks, and we assume here that DO is the limiting reactant for Fe redox reactions in the hyporheic zone, which is reasonable in AMD systems where solute-loading and acidic-water inputs are high. Mixing efficiency requires vertical characterization of DO gradients in the hyporheic zone with either nested wells or Minipoint sampling.^28^ We quantify ε as one minus the vertical DO gradient ΔO_2_/Δ*z*—where a well-mixed system would therefore have a small oxygen gradient across the vertical profile of the hyporheic zone (Figure 1B) and a poorly mixed system would have a high oxygen gradient across the vertical profile of the hyporheic zone (Figure 1A)—scaled by the mass transfer rate in the hyporheic zone α and porosity *n*
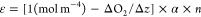
4where the mass transfer rate, α [s^–1^], quantifies the rate of movement of solutes between more-mobile and less-mobile pore space and can be estimated from breakthrough curve data using transient-storage models (i.e., OTIS).^29^ Porosity *n* [unitless] scales ε to the void space in porous sediments. We propose that the overall HRP in the hyporheic zone is a function of chemical favorability in terms of Gibbs free energy Δ*G* [kilojoules (kJ) mol^–1^], mixing efficiency ε, and area of the hyporheic zone (HZA, [m^2^]) such that
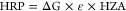
5This energy flux makes sense conceptually and estimates the capacity of the hyporheic zone to conduct biogeochemical work at a given time, delineating both physical and chemical controls of hyporheic zone function. HRP treats the hyporheic zone as a bioreactor to estimate the favorability for nutrient cycling, contaminant transformation, and other redox-sensitive reactions. HRP compliments existing metrics that quantify the relative importance of transient storage, reaction time scales, and transport time scales, and we compare HRP to two familiar tools, the Damkohler number and Reaction Significance Factor, in Supporting Information.

Conceptually, in ideal streams with a well-connected hyporheic zone, ΔO_2_/Δ*z* should be low, α high, and porosity abundant, which would expand the physical capacity of the hyporheic zone to promote redox reactions and increase HRP. Conversely, in systems with poor connection between the hyporheic zone and stream, ΔO_2_/Δ*z* should be high and mass transfer rates low, with potentially low porosity and, thus, lower HRP. Mixing efficiency (ε) scaled by hyporheic zone area parametrizes the physical capacity of the hyporheic zone to promote redox reactions. Δ*G* estimates thermodynamic potential for a given chemical reaction, and the cumulative HRP estimates overall energetic flux with units of KJ m^–2^ s^–1^. We formulate the reactions here such that a positive HRP indicates thermodynamic favorability for Fe oxidation (eq 1) or Fe-oxide precipitation (eq 2, 3) in the hyporheic zone, which would create a sink as Fe^2+^ oxidizes to Fe^3+^ and precipitates as Fe (oxy)hydroxides. Higher HRP values are indicative of geochemical and physical conditions that more strongly favor these Fe sink reactions. Conversely, a negative HRP indicates favorability for Fe reduction or metal oxide dissolution, which could create source conditions for metal(loid) release to streams as Fe^3+^ reduces to Fe^2+^ or Fe oxides to dissolve, increasing Fe concentrations in the water column. Similarly, more negative values are indicative of hyporheic zone conditions that more strongly favor Fe reduction and mineral dissolution.

Here, we calculate HRP_Fe_ for the three Fe reaction pathways in two field systems with different groundwater-streamwater connectivity, Mineral Creek and Cement Creek in the Bonita Mining District in Silverton, Colorado, USA, now a Superfund site composed of 48 former mines and mining structures. Mineral Creek is a well-connected stream with moderate Fe loading, and Cement Creek is poorly connected with high metal loading. We calculate HRP_Fe_ varying flow and redox conditions with surface water and nested well data from Hoagland et al.^14^ collected during high flow and low flow. More details on study sites, geochemical sampling, tracer studies, and Fe speciation are included in Supporting Information.

## Results and Discussion

Differing SW-GW exchange rates, mixing efficiency, and hydrologic connectivity drive increases in HRP_Fe_ in Mineral Creek but limit changes in HRP_Fe_ in Cement Creek as stream discharge increases (Table 1). Mineral Creek hyporheic sediments flip from an Fe sink (positive HRP) at high flow to an Fe source (negative HRP) at low flow. Stream discharge in Mineral Creek increases by an order of magnitude due to snowmelt, increasing bed mobility and mobilizing colloidal Fe,^14^ further contributing to Fe export and sink conditions delineated by HRP_Fe_. Physical flushing of Fe colloids and reductive dissolution likely help prevent pore clogging and improve the mixing efficiency of hyporheic-zone sediments in Mineral Creek. In contrast, in Cement Creek, solute reactions are limited by a disconnected hyporheic zone despite favorable hyporheic-zone biogeochemistry to promote Fe cycling. Mixing efficiency is over an order of magnitude lower in Cement Creek relative to Mineral Creek (Table 1) and has minimal changes across high- and low-flow conditions investigated here. These physical limitations, likely from ferricrete and other Fe-oxide precipitation, limit SW-GW exchange and minimize HRP_Fe_ despite chemical favorability for Fe-precipitation reactions in Cement Creek.

**Table 1:** HRP_Fe_ for Three Reaction Pathways in Two Streams with Varying Fe Concentrations under High- and Low-Flow Conditions^a^

Parameter	Mineral Cr. High Flow	Mineral Cr. Low Flow	Cement Cr. High Flow	Cement Cr. Low Flow
Fe^2+^ (μM)**	0.26	0.001	30	220
Fe^3+^ (μM)**	0.003	0.08	380	2.2
O_2_ (mM)*	0.31	0.30	0.27	0.26
O_2_ (mM)**	0.20	0.27	0.24	0.12
O_2_ (mM)***	0.28	0.28	0.05	0.07
SO_4_^2−^ (mM)**	0.32	2.13	4.36	5.40
pH**	6.50	6.50	3.90	4.00
ΔO_2_ (mM)	0.03	0.02	0.22	0.19
ΔZ (m)	0.7	0.7	0.53	0.53
ΔO_2_/ΔZ (mM m^–1^)	0.04	0.03	0.42	0.36
Porosity n (−)	0.35	0.35	0.35	0.35
Mass Transfer Rate α (s^–1^)	0.005	0.004	0.001	0.001
Hyporheic Area HZA (m^2^)	1.8	0.6	0.4	0.1
Damkohler Number (DaI)	0.9	2.9	0.1	0.1
Mixing Efficiency ε	1.7 × 10^–3^	1.4 × 10^–3^	2.2 × 10^–4^	2.4 × 10^–4^
Stream Width (m)	8.1	5.0	6.7	5.6
Stream Area (m^2^)	2.2	0.86	1.6	0.62
Discharge (m^3^/s)	2.2	0.12	1.3	0.18
Initial Oxidation Δ*G*_r_ (kJ/mol)	64	–22	54	120
Schwertmannite Δ*G*_r_ (kJ/mol)	310	380	230	150
Ferrihydrite Δ*G*_r_ (kJ/mol)	42	50	27	17
**Initial Oxidation HRP (kJ m**^**-2**^**s**^**-1**^**)**	0.19	–0.02	0.005	0.003
**Schwertmannite HRP (kJ m**^**-2**^**s**^**-1**^**)**	0.95	0.31	0.02	0.003
**Ferrihydrite HRP (kJ m**^**-2**^**s**^**-1**^**)**	0.13	0.04	0.002	0.0004

a Asterisks indicate sampling locations in nested wells where * indicates surface water, ** indicates shallow groundwater (depths = 0.20–0.28 m bgs), and *** indicates deeper groundwater (depths = 0.58–0.68 m bgs).

Mixing efficiency in Mineral Creek responds dynamically to changes in flow, while mixing efficiency in Cement Creek remains relatively constant across different flow conditions (Table 1). Mixing efficiency in Mineral Creek increases by 30% at high flow, and the hyporheic zone area more than doubles due to higher discharge in the stream. While mobile streambeds decrease the depth of hyporheic flow paths and may influence hyporheic area,^30^ the wetted perimeter of the stream increases by ∼3x at high flow relative to low flow in both streams (Table 1), and hyporheic-area estimates from numerical modeling in OTIS are more sensitive to wetted perimeter than water velocity in this case.^14,31^ These increases in mixing efficiency show that the hyporheic zone is larger and more connected at high flow rates, promoting Fe-redox-cycling reactions. While the hyporheic zone area increases at high flow in Cement Creek, mixing efficiency remains nearly constant, maintaining a strong vertical DO gradient. Thus, mixing efficiency and physical structure of the hyporheic zone limit Fe cycling in Cement Creek. These differences in hydrologic connectivity drive contrasts in hyporheic zone geochemistry between Mineral Creek and Cement Creek.

Mineral Creek and Cement Creek have disparate biogeochemical conditions across the various flow conditions investigated that may be controlled by SW-GW connectivity.^14^ In Mineral Creek, the dominant aqueous Fe species in shallow hyporheic water shift from Fe^3+^ at low flow to Fe^2+^ at high flow. Conversely, in Cement Creek Fe^3+^ is the predominant aqueous Fe species at high flow, and Fe^2+^ becomes the predominant aqueous species at low flow. In Mineral Creek, initial oxidation favorability (Δ*G*) shifts from an Fe sink, where precipitation of Fe^2+^ into Fe oxides are predicted at high flow, to an Fe source, where reductive dissolution of Fe^3+^ to Fe^2+^ is predicted at low flow (Table 1). Schwertmannite precipitation was favored across both high and low flows in Mineral Creek and Cement Creek and is more thermodynamically favorable than ferrihydrite precipitation, suggesting that schwertmannite is the initial Fe-oxidation mineral product in these acidic, sulfate-rich hyporheic sediments. Chemical favorability for initial oxidation and schwertmannite and ferrihydrite precipitation are positive and stay relatively constant as flow changes in Cement Creek (Table 1). However, limited SW-GW connectivity prevents these reactions from proceeding, despite sufficient chemical favorability.

Shifting between Fe source and sink may have implications for Fe cycling in Mineral Creek, where Fe is stored in Mineral Creek sediments via mineral precipitation or flushed downstream by bedform movement at high flow, and dissolution occurs at low flow, refreshing the capacity of the hyporheic zone to continue supporting Fe precipitation reactions on an annual time scale. Source-sink reversals maintain sediment porosity and permeability and minimize pore clogging of hyporheic sediments, supporting continued hyporheic-zone function over hydrogeologic time scales. This snowmelt-driven refreshment of hyporheic sediments in alpine watersheds is dependent on snow accumulation, snowmelt timing, and other factors that display large interannual variability and may be impacted by future climate.^24,32^ In Cement Creek, there is an imbalance between high concentrations of Fe loading into the stream and low HRP, leading to Fe-oxide and ferricrete precipitation that minimizes the mixing efficiency and limits SW-GW exchange. There is no source-sink transition observed under the high-flow and low-flow conditions investigated. Over hydrogeologic time scales, this imbalance of Fe loads relative to HRP_Fe_ causes sediment clogging that limits Cement Creek’s ability to mediate biogeochemical reactions and results in diminished HRP_Fe_. In short, Mineral Creek is well-connected and flips between the Fe sink and source as flow increases, which refreshes the sediment and prevents pore clogging from Fe mineral precipitation (Table 1). Cement Creek is limited by mixing efficiency, with solutes unable to react with each other due to ferricrete precipitation and other physical limitations despite favorable Δ*G* for reactions.

In summary, HRP delineates the physical and biogeochemical controls of solute fate and transport, demonstrated here for Fe fate and transport in two streams with varying hydrologic connectivity. The HRP framework integrates geochemical favorability (i.e., Δ*G*) with physical constraints from SW-GW interactions and the hyporheic zone area to define the reaction potential in the hyporheic zone, making it broadly applicable to pollution problems in stream systems. This framework addresses a long-standing research gap in delineating source-sink dynamics of the hyporheic zone and is a simple model that can be calculated with minimal data collection, and in many cases, data may already exist for preliminary calculations. It has particular relevance for diffuse-source pollution problems, such as acid-mine drainage, as it quantifies the reaction favorability for metal fate and transport across space and time. Using HRP, we find that Mineral Creek supports Fe-redox-cycling reactions in a well-connected hyporheic zone and suggest that in Cement Creek, sediment remediation could be used to improve mixing efficiency and SW-GW connectivity to decrease physical limitations to HRP_Fe_. However, restoration of draining mines may also need to be completed to limit metal loads into Cement Creek to levels that can be processed by the stream. Because the HRP framework integrates geochemical favorability (i.e., Δ*G*) with physical constraints from SW-GW interactions and the hyporheic zone area to define the reaction potential in the hyporheic zone, it can be expanded for other reactions where solute gradients related to hyporheic zone conditions drive reaction networks. While we developed this framework for Fe, it is transferrable to other redox-sensitive environmental pollutants, such as nitrate, sulfate, organic compounds, and uranium. This new approach quantifies source-sink dynamics of solutes in the hyporheic zone and has relevance to hydrologic, environmental-engineering, and water-resources communities. Quantifying event-scale metal fluxes and developing predictive tools to assess source-sink dynamics represent a step forward in quantifying and informing fate and transport to delineate biogeochemical and physical controls of redox cycling and thus our understanding of hyporheic zone function. In future work, we seek to quantify HRP in alpine streams with differing hydrologic and biogeochemical conditions at a higher temporal and spatial resolution.

## Supporting Information Available

Details on application field sites, geochemical sampling, tracer studies, Fe-speciation methods, and comparisons of HRP to existing metrics (PDF)
