# Supplementary material for: Hyporheic Reaction Potential: A Framework for Predicting Reach Scale Solute Fate and Transport
Source: Environ Sci Technol Lett. 2024 May 6;11(6):586–90. doi: 10.1021/acs.estlett.4c00035 (PMC11171444; doi:10.1021/acs.estlett.4c00035)
Supplement: Supplementary file 1 — ez4c00035_si_001.pdf [file ez4c00035_si_001.pdf]

Hyporheic reaction potential: A framework for predicting reach scale solute fate and transport

Supporting Information

Kenneth Swift Bird[1]*, Alexis Navarre-Sitchler[1,2], Kamini Singha[1,2]

[1]Hydrologic Science and Engineering Program, Colorado School of Mines, Golden, CO 80401

[2]Geology and Geological Engineering Department, Colorado School of Mines, Golden, CO 80401

*Corresponding author: kswiftbird@mines.edu

Supporting Information

Application Field Sites

Cement Creek is a headwaters tributary of the Animas River with a drainage area of approximately 24.6 km^2 located north of Silverton, Colorado (Figure SI-1). Metal(loid)-enriched water flows into Cement Creek via surface runoff from legacy mines, tailings piles, mine structures, natural groundwater flow paths, and via discharge from naturally occurring Fe fens. The Gold King Mine spill, an infamous release of over 11,000 m^3 of AMD, occurred into Cement Creek in 2015. Cement Creek is composed of metal(loid)-rich, low pH water, and ferricrete precipitation (cemented Fe-(oxy)hydroxides) is prominent in the streambed. Ferricrete thickness is spatially variable and limits SW-GW connectivity in some reaches of the watershed as it forms a relatively impermeable boundary[1]. In recent work, Fe concentrations in Cement Creek ranged from 0.1-14.2 mg/L[2]. We chose Cement Creek as a study site due to its high metal(loid) concentrations and low SW-GW connectivity, making it an ideal end member to study how event-based metal(loid) cycling responds to snowmelt and storm events in a poorly connected system.

Mineral Creek is also a headwaters tributary of the Animas River, located west of Silverton, Colorado (Figure SI-1). Mineral Creek has a drainage area of 23.1 km^2 and also flows through the Silverton Caldera. Remediation work was completed in this area in 2003, significantly limiting discharge of metal(loid)-enriched water to Mineral Creek[3]. Fe concentrations ranged from 0.1-0.3 mg/L in a recent study[1]. Ferricrete precipitation also occurs in Mineral Creek but is much less extensive than in Cement Creek. Mineral Creek is selected due to hydrologic, climactic, and geological similarities with Cement Creek, while maintaining good SW-GW connectivity with low to moderate metal(loid) concentrations in the stream and several proximal metal(loid) sources.

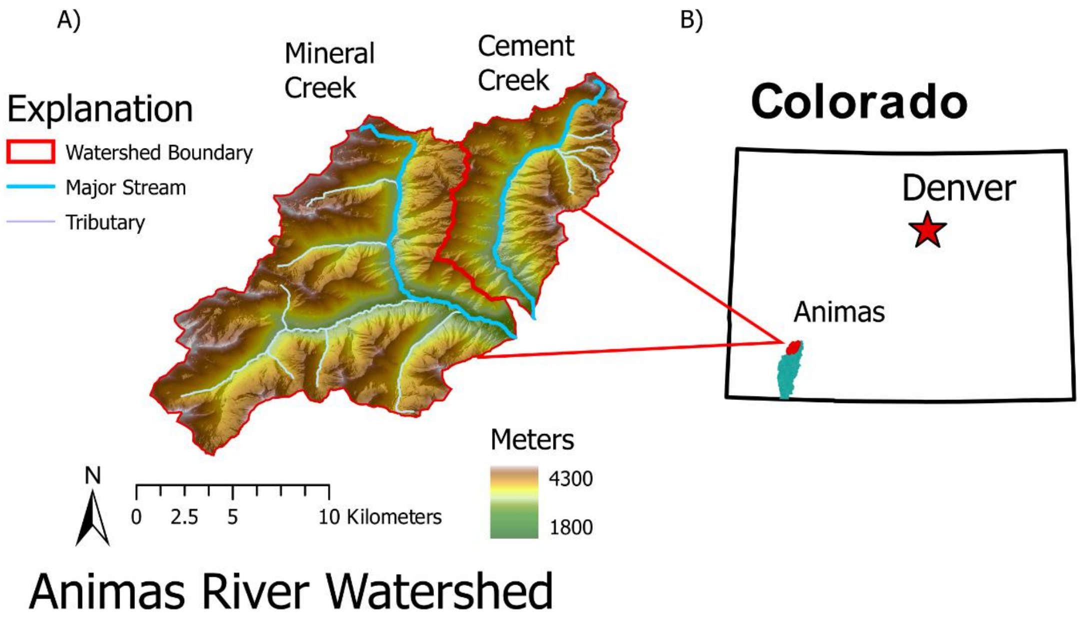

Figure SI-1: Watershed maps of Mineral Creek and Cement Creek near Silverton, CO showing A) Mineral Creek and Cement Creek watershed map detailing hydrology, topography, and elevation at the HUC12 scale and B) regional extent of the Animas River headwaters in southwestern CO, USA.

Geochemical Sampling, Tracer Study, and Fe Speciation Methods

Groundwater samples were collected in three well nests in each stream. These well nests were installed 0.28, 0.44, and 0.58 m deep in Cement Creek and 0.20, 0.40, and 0.68 m deep in Mineral Creek to measure redox properties and chemical concentrations across a vertical gradient in the hyporheic zone. Sampling was conducted at high flow and low flow in each stream, which were assumed to be hydrologic end members. Oxygen gradients were calculated using the difference in DO concentrations between surface water and the deepest groundwater well, and shallow groundwater chemistry (0.20-0.28 m bgs) was used to estimate thermodynamic potential of Fe dissolution and precipitation reactions in the hyporheic zone.

We defined the vertical extent of the hyporheic zone as the area around the stream with active exchange using tracer studies paired with electrical resistivity imaging from previous work[1,2] as well as a measurable DO gradient between surface water and nested groundwater wells. Shallow wells in both Mineral Creek and Cement Creek were within the redox front observed in the hyporheic zone based on nested-well DO concentrations. HRP represents a snapshot in time, so chemical parameters are all from the same sampling time for each calculation. While spatial heterogeneity of redox properties in the hyporheic zone exists, we are assuming that the radius of influence from sampling nested wells captures redox processes across a representative elementary volume that is appropriate to characterize local-scale hyporheic processes. However, we note that small-scale changes in oxygen gradients present at some sites may need to be captured by Minipoint sampling[4], which is easier to install and interpret to measure in low-gradient, sandy streams.

Fe^{2+} was measured directly in the field via colorimetry with a field spectrophotometer (HACH DR1900) using 1,10-phenanthroline reagent. However, in all but one sampling event (Cement Creek at high flow), the Fe^{2+} was above the upper detection limit of the method (3 mg/L), indicating high concentrations of dissolved Fe^{2+}. In samples where dissolved Fe^{2+} was above the field spectrophotometry detection limit, we estimated the dissolved Fe^{2+} concentration by calculating the equilibrium ratio of Fe^{2+} and Fe^{3+} from the total Fe concentration at an Eh of 0.70 V, the Eh calculated from the Fe^{2+}/Fe^{3+} concentration in the Cement Creek at high flow sample. Since the concentration of Fe^{2+} was lowest in this sample, we assumed that Eh = 0.70 V represents a maximum Eh value for samples where Fe^{2+} was at detectable concentrations.

Comparison of HRP to Existing Metrics

HRP compliments the familiar Damkohler number, a unitless parameter that describes the relative importance of transient storage and advective velocity[5]:

$$\mathrm{DaI} = \frac{\alpha\left(1+\frac{A}{A_S}\right)L}{u} \qquad \text{(SI-1)}$$

where A is stream area, A_S is storage area, L is reach length, and u is stream velocity. DaI predicts if streams will be transport limited (DaI > 1) or exchange limited (DaI < 1). These DaI formulations are broadly applicable and have been used to describe physical and chemical processes driving solute transformation in stream systems[6–8]. For example, Da was ~1 at high flow in Mineral Creek (Table 1), which supports a balance between exchange and advective time scales that enhances hyporheic zone capacity for biogeochemical reactions.

HRP expands beyond this idea by integrating thermodynamic favorability (Δ G), mixing efficiency (ε), and hyporheic zone area to estimate the hyporheic zone's potential to support redox transformations for a given solute. It delineates chemical and physical governing processes for solute exchange and geochemical reactions to estimate overall potential for a given geochemical reaction under specific hyporheic zone conditions. While DaI ~1 in Mineral Creek, there were biogeochemical favorability for initial Fe-oxidation, strong biogeochemical favorability for schwertmannite precipitation, and slight thermodynamic favorability for ferrihydrite oxidation that cannot be accounted for by DaI. During low flow, DaI shifted to transport-limited conditions, there was a redox reversal where Fe-reductive dissolution became favored, and mixing efficiency decreased by 30%, impeding the capacity of the hyporheic zone to support biogeochemical reactions and reversing source - sink dynamics. Cement Creek was exchange-limited in DaI, and had a low ε at both high and low flow, highlighting that solute interaction was limited even if sufficient chemical gradients as shown in HRP calculations (Table 1) were present to support biogeochemical reactions.

Another metric to assess the capacity of the hyporheic zone to support redox reactions is the Reaction Significance Factor[9]:

$$R_s = \frac{\lambda_{hz}\tau_{hz}L_c}{L_s}. \qquad \text{(SI-2)}$$

This unitless term relates reaction timescales (λ_{hz}), hyporheic residence times (τ_{hz}), characteristic reach length (L_c), and storage zone flowpath length (L_s) to estimate the relative importance of the hyporheic zone in mediating redox reactions. R_s calculates the relative importance of reaction timescales and mass-transfer timescales, similar to the Damkohler number. Higher R_s values indicate greater capacity of the hyporheic zone to mediate redox reactions. For the hyporheic zone to be an important contributor to watershed-scale solute mass balances, R_s must be greater than 0.2[9]. R_s requires a combination of data from tracer studies and geochemical measurements, which is similar to our HRP term. HRP differs from R_s in that it explicitly parameterizes thermodynamic favorability and oxygen gradients in the hyporheic zone to estimate reaction favorability in energy units [KJ m^{-2} s^{-1}]. R_s requires an in-stream tracer test, and sampling of tracer concentrations in hyporheic water and groundwater during that tracer test to estimate reaction timescales[4], which were not available for our dataset.

References

(1) Hoagland, B.; Navarre-Sitchler, A.; Cowie, R.; Singha, K. Groundwater–Stream Connectivity Mediates Metal(Loid) Geochemistry in the Hyporheic Zone of Streams Impacted by Historic Mining and Acid Rock Drainage. *Frontiers in Water* **2020**, *2*.

(2) Rickel, A.; Hoagland, B.; Navarre-Sitchler, A.; Singha, K. Seasonal Shifts in Surface Water-Groundwater Connections in a Ferricrete-Impacted Stream Estimated from Electrical Resistivity. *Geophysics* **2021**, *86* (5), WB117–WB129. https://doi.org/10.1190/geo2020-0599.1.

(3) Walton-Day, K.; Paschke, S. S.; Runkel, R. L.; Kimball, B. A. Using the OTIS Solute-Transport Model to Evaluate Remediation Scenarios in Cement Creek and the Upper Animas River.

(4) Harvey, J. W.; Böhlke, J. K.; Voytek, M. A.; Scott, D.; Tobias, C. R. Hyporheic Zone Denitrification: Controls on Effective Reaction Depth and Contribution to Whole-Stream Mass Balance. *Water Resources Research* **2013**, *49* (10), 6298–6316. https://doi.org/10.1002/wrcr.20492.

(5) Bahr, J. M.; Rubin, J. Direct Comparison of Kinetic and Local Equilibrium Formulations for Solute Transport Affected by Surface Reactions. *Water Resources Research* **1987**, *23* (3), 438–452. https://doi.org/10.1029/WR023i003p00438.

(6) Briggs, M. A.; Day-Lewis, F. D.; Ong, J. B. T.; Curtis, G. P.; Lane, J. W. Simultaneous Estimation of Local-Scale and Flow Path-Scale Dual-Domain Mass Transfer Parameters Using Geoelectrical Monitoring. *Water Resources Research* **2013**, *49* (9), 5615–5630. https://doi.org/10.1002/wrcr.20397.

(7) Oldham, C. E.; Farrow, D. E.; Peiffer, S. A Generalized Damköhler Number for Classifying Material Processing in Hydrological Systems. *Hydrology and Earth System Sciences* **2013**, *17* (3), 1133–1148. https://doi.org/10.5194/hess-17-1133-2013.

(8) Zarnetske, J. P.; Haggerty, R.; Wondzell, S. M.; Bokil, V. A.; González-Pinzón, R. Coupled Transport and Reaction Kinetics Control the Nitrate Source-Sink Function of Hyporheic Zones. *Water Resources Research* **2012**, *48* (11). https://doi.org/10.1029/2012WR011894.

(9) Harvey, J. W.; Fuller, C. C. Effect of Enhanced Manganese Oxidation in the Hyporheic Zone on Basin-Scale Geochemical Mass Balance. *Water Resources Research* **1998**, *34* (4), 623–636. https://doi.org/10.1029/97WR03606.
